# Evaluating volumetric modulated arc therapy planning efficiency and dosimetric impact on non-contoured organ at risk in whole brain radiotherapy: a focus on oral cavity dose distribution

**DOI:** 10.1590/1806-9282.20250188

**Published:** 2025-12-05

**Authors:** Hatice Halis, Uğur Akbayırlı

**Affiliations:** 1 Sakarya University Training and Research Hospital, Radiation Oncology Clinic – Sakarya, Turkey.; 2 Sakarya University of Applied Sciences, Biomedical Technologies Application and Research Center – Sakarya, Turkey.

**Keywords:** Brain neoplasms, Secondary, Intensity-modulated, Radiotherapy planning, Mouth, Radiation effects

## Abstract

**OBJECTIVE:**

The aim of this study was to investigate the dosimetric outcomes and planning efficiency of volumetric modulated arc therapy compared to the field-in-field technique for whole brain radiotherapy, with a focus on the oral cavity as a newly defined organ at risk. The study also evaluates the impact of volumetric modulated arc therapy re-optimization on reducing oral cavity doses while ensuring target coverage.

**METHODS:**

Treatment plans for 20 technique for whole brain radiotherapy patients treated with volumetric modulated arc therapy were retrospectively analyzed and re-evaluated using field-in-field and volumetric modulated arc therapy re-optimization techniques. Dosimetric parameters, including D2%, D98%, V30(Gy), homogeneity index, and conformity index, were compared for the planning target volume. Mean and maximum doses to the oral cavity and bilateral lenses were assessed.

**RESULTS:**

Volumetric modulated arc therapy provided superior conformity (CI 0.82±0.07) and homogeneity (HI: 1.05±0.00) compared to field-in-field (CI 0.65±0.13, HI: 1.06±0.10) with significant differences (p<0.001). However, volumetric modulated arc therapy resulted in higher mean and maximum oral cavity and lens doses. Volumetric modulated arc therapy re-optimization significantly reduced oral cavity doses (p<0.001) while maintaining similar target coverage and dosimetric performance to volumetric modulated arc therapy.

**CONCLUSIONS:**

Volumetric modulated arc therapy improves dose conformity and homogeneity compared to field-in-field but increases doses to non-contoured organ at risk. Volumetric modulated arc therapy re-optimization effectively lowers oral cavity doses without compromising target coverage, underscoring the importance of identifying and addressing non-standard organ at risk. These findings highlight volumetric modulated arc therapy's clinical advantages, particularly in high-demand settings, while emphasizing the need for optimizing organ at risk to reduce toxicity.

## INTRODUCTION

Brain metastases (BM) are the most prevalent intracranial tumors in adults and the primary cause of neurological disorders stemming from systemic cancers^1^. Research indicates that neurological symptoms and the median overall survival of patients with BM can improve with whole brain radiotherapy (WBRT), extending survival from 1–2 months to 3–6 months^2,3^. As treatment techniques have advanced, patients with a favorable prognosis and limited disease are often treated with stereotactic radiosurgery. Nevertheless, WBRT continues to be a crucial treatment option for those with multiple BM or a poor prognosis^4^.

Recent clinical trials have shown that WBRT using the volumetric modulated arc therapy (VMAT) technique offers a more precise and uniform dose distribution for the entire brain while protecting certain critical organ at risk (OAR). Moreover, VMAT provides benefits such as shorter treatment times than traditional methods. In contrast, the widely used field-in-field (FIF) 3D conformal technique has a less optimal dose distribution but may lead to lower toxicities for distant normal tissues and non-contoured OAR^5^.

## METHODS

Notably, 20 WBRT patients previously treated with the VMAT technique at Sakarya Training and Research Hospital were randomly selected and enrolled in this retrospective study. All patients had a prior primary cancer diagnosis with multiple metastases to the brain. This study was approved by the Ethics Committee of Sakarya University Faculty of Medicine (Approval No. E-16214662-050.01.04-95063-238, dated January 13, 2022). Written consent was obtained from each patient for the study.

All patients were simulated in the supine position. Radon Head & Neck support cushions and thermoplastic masks were used for immobilization. Computed tomography (CT) simulation images (native, 120 kV, 80 mA, slice thickness 1.25 mm) were acquired using a GE Discovery RT (Fairfield, Connecticut, USA) machine. The CT simulation images were electronically transferred to the MIM 6.9 (Cleveland, OH, USA) contouring software for organ delineation. The whole brain was defined as the target structure, while both lenses were designated as OAR. The patient's outer contour was delineated by a single radiation oncologist, and the oral cavity was retrospectively contoured according to the consensus guidelines of Grégoire et al.^6,7^. For this study, the oral cavity was also contoured afterward for re-optimization in the retrospective evaluation^8,9^. Once the contouring was completed, the patient's CTs and structures were transferred to the Monaco 5.55 (Elekta AB, Stockholm, Sweden) treatment planning system (TPS). The whole brain planning target volume (PTV) for optimization was created by delineating the whole brain with a 0.3 cm isotropic margin in all directions.

### Treatment planning

For selected patients, previously planned and treated treatment plans with VMAT (VMAT-treated, VMATt) techniques were retrieved and replanned using the 3D conformal FIF technique and secondary VMAT re-optimization (VMATro) with the newly delineated oral cavity as an OAR.

FIF plans were created with two static fields at gantry angles of 90° and 270°, and consequently generated two to three sub-segments. All FIF plans were calculated using the collapsed cone (CC) calculation algorithm on the Monaco 5.55 TPS.

VMAT plans were created with full arcs (360° rotation) and two rotations (clockwise and counterclockwise) per plan. For calculation, the maximum number of control points was set to 360, the calculation grid size was 0.3 cm, and statistical uncertainties were 1.0%. All VMAT optimizations were calculated using the Monte Carlo (MC) calculation algorithm on the Monaco 5.55 TPS.

For all planning techniques, the treatment prescription for the whole brain PTV was set to deliver 30 Gy over the course of 10 fractions. All plans were normalized to ensure that 95% of the whole brain PTV was covered by 95% of the prescribed dose. All acceptable compliance criteria for the whole brain PTV and OARs in the plans are listed in Table 1.

**Table 1:** Dose volume histogram criteria of structures for treatment plans.

Structure parameter	DVH goal	Max acceptable criteria
PTV D98%	>2,850 cGy	>2,800 cGy
PTV D2%	<3,250 cGy	<3,300 cGy
Lens (individually) mean dose	<600 cGy	<1,000 cGy
Oral cavity	ALARA*	ALARA*

ALARA: as low as reasonably achievable; DVH: dose volume histogram; PTV: planning target volume.

* ALARA—indicating that doses should be minimized without compromising treatment efficacy.

In this study, before comparing the doses to OAR, we compared the dosimetric parameters of three radiotherapy techniques: FIF, VMATt, and VMATro. We focused on the PTV metrics D2%, D98%, V30, homogeneity index (HI), and conformity index (CI) to evaluate the treatment efficiency of each plan.

Additionally, planning and treatment times were compared for these techniques. For planning time comparison, we measured the time from selecting a new plan to the start of the final calculation. The final calculation time was excluded because VMAT plans were calculated using the MC method, whereas FIF plans used the CC method for each sub-segment. We used template-based planning for all techniques, eliminating the need for changes to beam parameters and IMRT constraints. The time for oral cavity contouring was also considered. For treatment time comparison, the calculated final monitor units (MU) were also noted.

Lastly, all three versions of plans for 20 patients were ­created on an Octavius 4D (PTW, Freiburg, Germany) phantom in Monaco 5.55 and irradiated on a Versa HD treatment machine. The patient plan quality assurance (QA) results were analyzed using Verisoft (PTW, Freiburg, Germany) software to detect possible plan calculation issues.

### Statistical analysis

All statistical analyses were done with SPSS version 21 (IBM, Armonk, New York, USA) software. The normality of data was assessed using the Shapiro-Wilk test. Depending on the distribution, either the paired t-test or the Wilcoxon signed-rank test was used for paired comparisons. In evaluation, p<0.001 were considered to be statistically significant.

## RESULTS

All three plan results were averaged for 20 cases. For the target dose distribution comparison, whole brain PTV results were ­examined. All mean results for these metrics can be shown in Table 2.

**Table 2:** Target dose distribution metrics for three different treatment planning techniques.

Target metric	FIF mean	VMATt mean	VMATro mean	Statistical significance
D2% of PTV (cGy)	3,119.86±21.70	2,984±62.94	3,129.53±14.66	p>0.005
D98% of PTV (cGy)	2,852.88±49.51	2,859.38±137.89	2,853.28±54.47	p>0.005
V30(Gy) of PTV (%)	69.12%±13.14	83.92%±7.41	78.36%±7.89	FIF vs. VMATt: **p<0.001** VMATt vs. VMATro: p>0.005
Homogeneity index (HI)	1.06±0.10	1.05±0.00	1.06±0.013	p>0.001
Conformity index (CI)	0.65±0.13	0.82±0.07	0.78±0.079	FIFvs. VMATt/VMATro: **p<0.001**; VMATt vs. VMATro: p>0.001
Oral cavity mean dose (cGy)	151.33±30.53	381.87±239.26	104.25±19.97	FIF vs. VMATt: **p=0.002** FIF vs. VMATro: **p<0.001** VMATt vs. VMATro: **p<0.001**
Oral cavity maximum dose (cGy)	691.87±201.14	1,202.60±436.69	256.15±71.05	FIF vs. VMATt: **p=0.002**; FIF vs. VMATro: **p<0.001**; VMATt vs. VMATro: **p<0.001** FIF vs. VMATro: **p<0.001**; VMATt vs. VMATro: **p<0.001**
Right lens mean dose (cGy)	509.36±22.90	520.71±17.71	342.24±56.57	FIF vs. VMATro: **p<0.001** VMATt vs. VMATro: **p<0.001**
Left lens mean dose (cGy)	525.63±67.01	530.08±16.35	328.37±48.59	FIF vs. VMATro: **p<0.001**; VMATt vs. VMATro: **p<0.001**

FIF: field-in-field; VMATt: volumetric modulated arc therapy-treated; PTV: planning target volume; VMATro: volumetric modulated arc therapy re-optimization. Bold values indicate statistically significant differences (p<0.001).

The D2% of PTV coverage values were 3,119.86±21.70 cGy for FIF, 2,984±62.94 cGy for VMATt, and 3,129.53±14.66 cGy for VMATro. VMATro exhibited the highest D2% value, indicating a slightly higher dose to the top 2% of the volume compared to FIF and VMATt, but this result shows no statistical significance compared to the other techniques.

Although VMATro showed a marginally higher D2% (3,129.53 cGy) compared to FIF and VMATt, this value remains within our institutional tolerance limit of <3,300 cGy. Therefore, the observed increase is considered clinically acceptable.

For D98% of PTV coverage, the values were ­ for FIF, 2,859.38±137.89 cGy for VMATt, and 2,853.28±54.47 cGy for VMATro. Due to PTV coverage normalization, all techniques provided similar D98% values, suggesting ­comparable coverage of the target volume, though VMATt displayed slightly higher variability.

The V30(Gy) of PTV values were 69.12%±13.14% for FIF, 83.92%±7.41% for VMATt, and 78.36%±7.89% for VMATro. VMATt achieved the highest percentage of the volume receiving 30 Gy, indicating superior coverage at this dose level compared to FIF and VMATro, and this result is statistically significant (p<0.001) over FIF but not for VMATro.

The HI values were 1.06±0.10 for FIF, 1.05±0.00 for VMATt, and 1.06±0.013 for VMATro. All techniques demonstrated similar homogeneity, with VMATt showing the least variability.

The CI values were 0.65±0.13 for FIF, 0.82±0.07 for VMATt, and 0.78±0.079 for VMATro. VMATt provided the highest conformity, indicating better alignment of the dose distribution with the target volume compared to FIF and VMATro. The better CI results for both VMAT techniques are statistically significant (p<0.001) against the FIF technique, but there is no statistical difference between the VMAT techniques.

**Figure 1:**
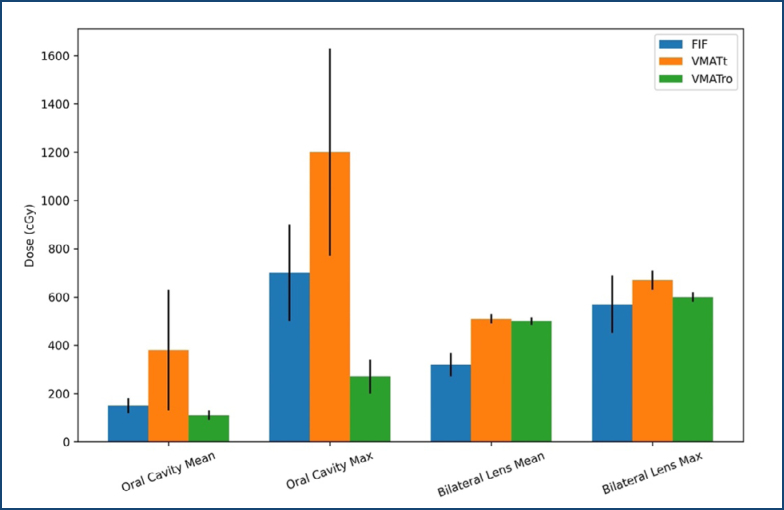
Organ at risk doses for three treatment planning techniques.

For the three planning techniques, the mean and maximum OAR values for 20 patients were averaged and are presented in Figure 1, in which the black lines represent standard deviations. Regarding the well-known treatment-related OAR, the bilateral lens exhibited a mean dose where VMATt and VMATro showed similar levels, both significantly higher than FIF (p<0.001). This pattern was consistent for the bilateral lens maximum dose, with VMATt and VMATro again ­delivering comparable doses that exceeded those of FIF; however, these results did not reach statistical significance at this time.

As a newly contoured OAR, the oral cavity received the highest mean dose from VMATt, followed by FIF and VMATro. In addition to that, the oral cavity's maximum dose was significantly higher with VMATt compared to FIF and VMATro. Besides that, VMATro has the lowest values for both mean and maximum oral cavity doses compared to VMATt and FIF. All these results are statistically significant (p<0.001).

Average planning times in Monaco 5.51 TPS for one individual planner were 7.2±0.3 min for VMAT (VMATt and VMATro) and 21.4±0.7 min for FIF. There was a statistically significant time difference (p<0.001) in favor of VMAT planning. In contrast, oral cavity OAR contouring took an average of 11.0±0.7 min by a single radiation oncologist.

For treatment time illustration, the final calculated average MUs were 464 for FIF, 811 for VMATt, and 872 for VMATro. FIF had significantly fewer MUs (p<0.001) compared to both VMATt and VMATro. However, there was no statistical significance between the differences in the VMAT techniques.

All different plan techniques were irradiated on the Octavius 4D phantom and evaluated using Verisoft software as part of the patient QA procedure. All QA results showed over 95% agreement with the total dose distribution of TPS-calculated plans, meeting the 3% DD and 3 mm DTA gamma index passing rate criteria.

## DISCUSSION

This study examined the dosimetric results and planning efficiency of the VMAT technique compared to the conventional FIF technique for WBRT, and particularly highlighted the oral cavity as an OAR that is often overlooked in WBRT dose distribution assessments. The study cohort consisted of 20 patients, which is a relatively small sample size and may limit the generalizability of the findings. The cohort included patients with a variety of primary cancer types and varying metastatic burden, reflecting the heterogeneity typical of WBRT populations as described by Tsao et al^4^. Previous research has shown that the VMAT technique generally provides better dose conformity and homogeneity to target volumes than the FIF technique^5^. The clinical significance of oral cavity dose reduction is supported by recent studies. Bansal et al. demonstrated a correlation between mean oral mucosa dose and the risk of Grade 3 mucositis in head and neck cancer patients treated with VMAT. In our cohort, the mean oral cavity dose with VMATro (~600 cGy) is well below the threshold associated with high-grade toxicity, suggesting a lower risk of severe mucositis and xerostomia. However, patient-reported outcomes were not collected in this study, which is a limitation^10^.

Oral complications, such as xerostomia and candidiasis, are common issues that can adversely impact patients ­following radiotherapy treatments in the oral cavity area. A further ­limitation is the lack of long-term follow-up data on oral complications such as mucositis and candidiasis. Previous studies, such as Vissink et al., have highlighted the importance of ­monitoring late effects after head and neck radiotherapy^11,12^. Reducing radiation dose to the oral cavity may improve patients’ quality of life^13^. The ability of VMAT to provide adequate target ­coverage while reducing the dose to the oral cavity may provide significant clinical benefit.

Our study highlights the importance of including the oral cavity as an uncontoured OAR. Re-optimized VMAT plans (VMATro) significantly reduced doses to the oral cavity while maintaining target coverage, potentially improving patient ­quality of life. Accurate OAR delineation and appropriate optimization are crucial for improved outcomes in modulated radiotherapy^14^. While VMAT offers planning benefits, its potential for increased late toxicities due to larger low-dose areas needs further investigation^15^. Our study's limited sample size cannot address this fully. Prospective studies with larger cohorts are essential, though the short median survival of WBRT patients may limit the feasibility of long-term follow-up, and also ­clinical validation with prospective studies assessing toxicity and patient-reported outcomes is necessary before drawing conclusions regarding clinical benefit.

Analysis of planning efficiency showed a significant time advantage for VMAT compared with FIF. This aligns with previous findings emphasizing the importance of efficient planning in busy clinical settings^16^. Although the inclusion of oral cavity contouring increased the time required for the VMAT workflow, the overall planning time for VMAT was still significantly shorter than for FIF. This time-saving benefit, together with the dosimetric advantages of VMAT, strongly supports its use in WBRT. Despite the advantages of VMAT, the FIF technique may still be preferred in certain clinical scenarios, such as palliative cases where rapid planning and lower lens doses are prioritized over dose conformity. This is particularly relevant for patients with limited life expectancy or when resources are tight.

It should be noted that different dose calculation algorithms were used for the two techniques: MC for VMAT and CC for FIF. This may introduce algorithm-dependent biases in dose estimation. Teoh et al. compared the accuracy of these algorithms and found that MC generally provides more accurate dose calculations, especially in heterogeneous regions^5^. Besides that, our volume of interest is mostly homogeneous brain tissue covered by the skull, and the effect of the calculation algorithm is supposed to be negligible.

In conclusion, VMAT, especially when re-optimized (VMATro), marks a significant improvement in WBRT. It enhances dose distribution and planning efficiency while lowering doses to critical OAR such as the oral cavity. These results highlight the necessity of personalized treatment planning and thorough OAR contouring to attain the best therapeutic results and reduce treatment-related side effects.

## Data Availability

The datasets generated and/or analyzed during the current study are available from the corresponding author upon reasonable request.

## References

[1] McKay MJ (2021). Brain metastases: increasingly precision medicine-a narrative review. Ann Transl Med.

[2] Li AY, Gaebe K, Jerzak KJ, Cheema PK, Sahgal A, Das S (2022;). Intracranial metastatic disease: present challenges, future opportunities. Front Oncol.

[3] Lima FM, Carvalho AL, Silva JC, Amorim R, Dellaretti M, Wendling-Henriques LA (2016). Treatment of brain metastases. Rev Assoc Med Bras (1992).

[4] Tsao MN, Rades D, Wirth A, Lo SS, Danielson BL, Gaspar LE (2012). Radiotherapeutic and surgical management for newly diagnosed brain metastasis(es): an American Society for Radiation Oncology evidence-based guideline. Pract Radiat Oncol.

[5] Teoh M, Clark CH, Wood K, Whitaker S, Nisbet A (2011). Volumetric modulated arc therapy: a review of current literature and clinical use in practice. Br J Radiol.

[6] Grégoire V, Evans M, Le QT, Bourhis J, Budach V, Chen A (2018). Delineation of the primary tumour Clinical Target Volumes (CTV-P) in laryngeal, hypopharyngeal, oropharyngeal and oral cavity squamous cell carcinoma: AIRO, CACA, DAHANCA, EORTC, GEORCC, GORTEC, HKNPCSG, HNCIG, IAG-KHT, LPRHHT, NCIC CTG, NCRI, NRG Oncology, PHNS, SBRT, SOMERA, SRO, SSHNO, TROG consensus guidelines. Radiother Oncol.

[7] Scoccianti S, Detti B, Gadda D, Greto D, Furfaro I, Meacci F (2015). Organs at risk in the brain and their dose-constraints in adults and in children: a radiation oncologist's guide for delineation in everyday practice. Radiother Oncol.

[8] Dean JA, Welsh LC, Gulliford SL, Harrington KJ, Nutting CM (2015). A novel method for delineation of oral mucosa for radiotherapy dose-response studies. Radiother Oncol.

[9] Stieb S, Mohamed ASR, He R, Zhu LL, McDonald BA, Wahid K (2021). Development and validation of a contouring guideline for the taste bud bearing tongue mucosa. Radiother Oncol.

[10] Bansal A, Bedi N, Kaur R, Singh G, Benipal RPS, Dangwal V (2023). Correlation of oral mucosa dose and volume parameters with Grade 3 mucositis, in patients treated with volumetric modulated arc radiotherapy for oropharyngeal cancer?. Jpn J Clin Oncol.

[11] Rajendra Santosh AB, Boyd D, Laxminarayana KK (2020). Clinical outline of oral diseases. Dent Clin North Am.

[12] Vissink A, Burlage FR, Spijkervet FK, Jansma J, Coppes RP (2003). Prevention and treatment of the consequences of head and neck radiotherapy. Crit Rev Oral Biol Med.

[13] Chen AM, Daly ME, Farwell DG, Vazquez E, Courquin J, Lau DH (2014). Quality of life among long-term survivors of head and neck cancer treated by intensity-modulated radiotherapy. JAMA Otolaryngol Head Neck Surg.

[14] Wright JL, Yom SS, Awan MJ, Dawes S, Fischer-Valuck B, Kudner R (2019). Standardizing normal tissue contouring for radiation therapy treatment planning: an ASTRO consensus paper. Pract Radiat Oncol.

[15] Gaspar LE, Mehta MP, Patchell RA, Burri SH, Robinson PD, Morris RE (2010). The role of whole brain radiation therapy in the management of newly diagnosed brain metastases: a systematic review and evidence-based clinical practice guideline. J Neurooncol.

[16] Pütz M, Wenz F (2012). Current strategies in radiotherapy of head and neck cancer. GMS Curr Top Otorhinolaryngol Head Neck Surg.

